# Is calcium deficiency the real cause of bitter pit? A review

**DOI:** 10.3389/fpls.2024.1383645

**Published:** 2024-06-24

**Authors:** Estanis Torres, Lee Kalcsits, Luís Gonzalez Nieto

**Affiliations:** ^1^ Institute of Agrifood Research and Technology (IRTA), Fruitcentre, Parck AgroBiotech, Lleida, Spain; ^2^ Tree Fruit Research and Extension Center, Washington State University, Wenatchee, WA, United States; ^3^ Department of Horticulture, Washington State University, Pullman, WA, United States; ^4^ School of Integrative Plant Sciences, Horticulture Section, New York State Agricultural Experiment Station, Cornell University, Geneva, NY, United States

**Keywords:** calcium disorders, physiological disorders, plant nutrition, plant growth regulators, postharvest, *Malus domestica*

## Abstract

Bitter pit is a disorder affecting the appearance of apples. Susceptibility is genetically controlled by both the cultivar and rootstock, with both environmental and horticultural factors affecting its severity and proportional incidence. Symptoms appear more frequently at the calyx end of the fruit and consist of circular necrotic spots, which take on a “corky” appearance visible through the peel. Bitter pit may develop before harvest, or after harvest, reducing the proportions of marketable fruit. In this review, current knowledge of the factors associated with the occurrence of bitter pit in apples is summarized and discussed along with their interactions with Ca uptake and distribution to fruit. This disorder has been previously linked with localized Ca deficiencies in fruit during its development. However, these relationships are not always clear. Even with over a century of research, the precise mechanisms involved in its development are still not fully understood. Additional factors also contribute to bitter pit development, like imbalances of mineral nutrients, low concentration of auxins, high concentration of gibberellins, changes in xylem functionality, or physiological responses to abiotic stress. Bitter pit remains a complex disorder with multiple factors contributing to its development including changes at whole plant and cellular scales. Apple growers must carefully navigate these complex interactions between genetics, environment, and management decisions to minimize bitter pit in susceptible cultivars. Accordingly, management of plant nutrition, fruit crop load, and tree vigor still stands as the most important contribution to reducing bitter pit development. Even so, there will be situations where the occurrence of bitter pit will be inevitable due to cultivar and/or abiotic stress conditions.

## 1 Introduction

**Figure 1:**
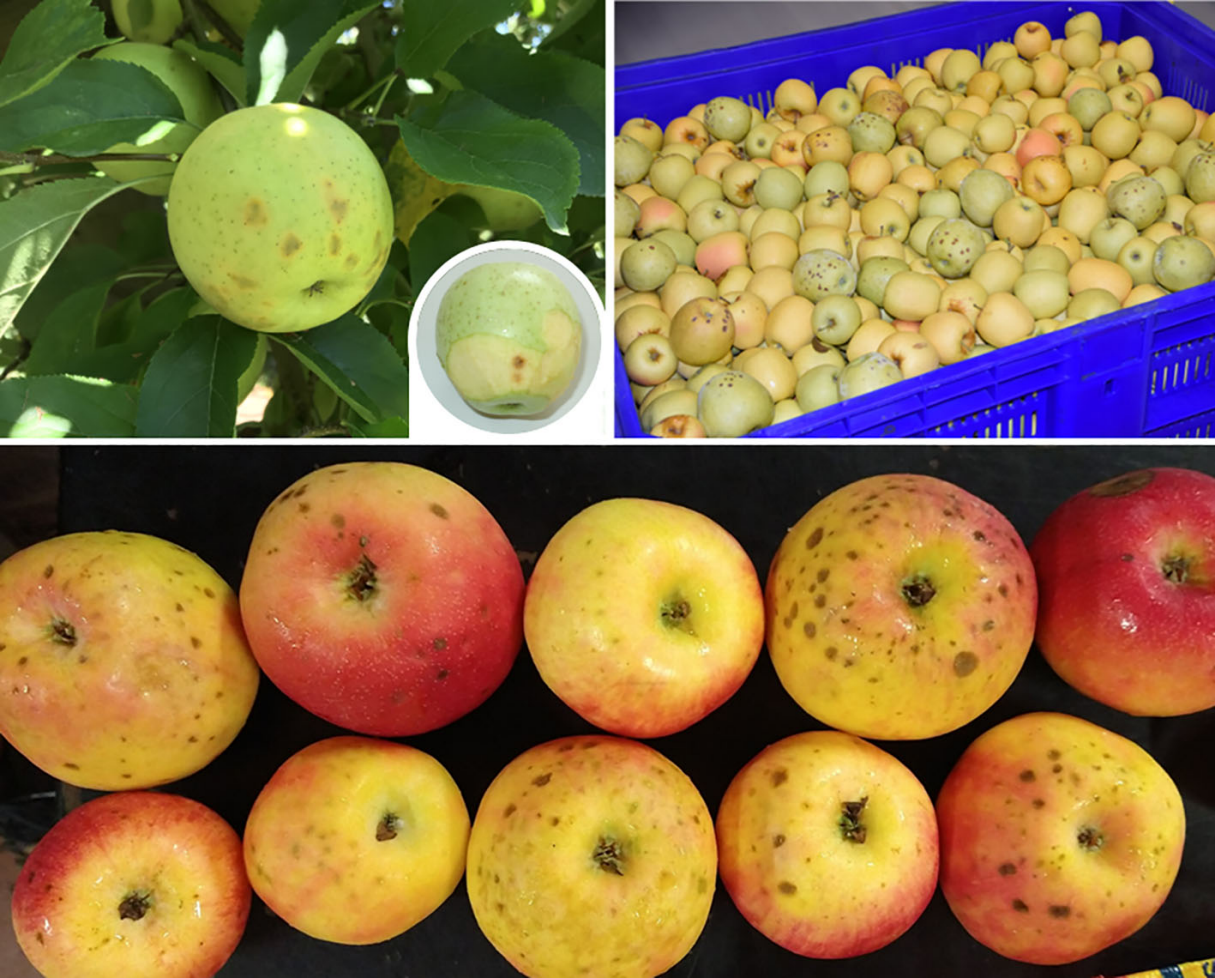
Bitter pit symptoms in ‘Golden’(above) and ‘Honeycrisp’ (below) apples.

Bitter pit is one of the most common physiological disorders for pome fruit, causing significant annual losses. Despite over a century of research, the exact causes of bitter pit are still not fully understood. Bitter pit symptoms manifest from cellular breakdown under the peel, causing necrotic and corky lesions generally concentrated at the calyx end of the fruit. These lesions are commonly darkened, dry, and spongy with a bitter taste (**Figure 1**). Bitter pit symptoms may appear on the tree or after harvest during the first month or two of storage (Serban et al., 2019). Since the identification of bitter pit as a disorder in the 1920s, numerous efforts have been made to explain the underlying physiological reasons behind its development. However, most of the publications have focused on describing the agronomic factors contributing to increased bitter pit incidence.

Traditionally, bitter pit has been most often associated with low calcium (Ca) content in the fruit (Smock and Van Doren, 1937; Van Stuivenberg and Pouwer, 1950; Yamazaki et al., 1968; Ferguson et al., 1979). Ca is taken up by the tips of actively growing roots and is transported through xylem tissue to transpiring tissues above ground including leaves and fruit (de Freitas and Mitcham, 2012; Miqueloto et al., 2014; Kalcsits et al., 2017). Uptake and transport of Ca are through the transpiration stream, and unlike many other nutrients, Ca is not phloem mobile and cannot be easily translocated from one organ to another when demands change (de Freitas and Mitcham, 2012). Ca deficiency can be due to low Ca availability or water stress, which results in low transpiration rates since Ca cannot be mobilized from older tissues and redistributed via phloem (Thomas and Adarsh, 2020). Therefore, localized Ca deficiencies like bitter pit commonly appear on the furthest ends of an organ to the xylem supply (Miqueloto et al., 2014). However, bitter pit is a complex process that involves not only the total input of Ca into the fruit but also a proper Ca^2+^ homeostasis at the cellular level, affecting different cellular fractions present in the plant (free Ca^2+^, pectates, phosphates, carbonates, and oxalates) and bitter pit development (de Freitas et al., 2010). The proportion of Ca pectate in cell walls is associated with the susceptibility of tissue to fungal and bacterial infections (Thomas and Adarsh, 2020), and together with Ca^2+^ accumulation into storage organelles, both fractions may have an important role in the bitter pit development as shown by de Freitas et al. (2010). Additionally, Ca can act as a secondary messenger for several metabolic processes (Xu et al., 2022), and its cytosolic levels must be precisely controlled since variation can trigger diverse metabolic responses and even lead the cell to death (Tang and Luan, 2017; Paiva, 2019). There is a clear relationship between the status of Ca^2+^ homeostasis inside cells and bitter pit. However, as we will present later, the mechanisms underlying bitter pit development still remain unclear.

The uptake and distribution of Ca in apples are interconnected with other nutrients, and an imbalance can also lead to the appearance of Ca disorders (Ludders, 1979; Ferguson and Watkins, 1983; Hopfinger et al., 1984; Pavičić and Miljcović, 1991; Amarante et al., 2013). Since many nutrients are phloem mobile, Ca relative to other nutrients is an important indicator of bitter pit risk (Ferguson and Watkins, 1983). However, additional factors have been identified that also contribute to its development, like variation in auxin concentration (Griffith et al., 2022; Huang et al., 2023), high concentration of gibberellins (Saure, 2005), changes in xylem functionality (Miqueloto et al., 2014), or physiological responses to abiotic stress (Mao et al., 2021). All these factors have been implicated in bitter pit development, but the relationships among them are still not well defined. In fact, most of these additional factors also contribute to fruit Ca content; hence, Ca deficiencies could possibly be parallel phenomena rather than direct causes of bitter pit in some cases.

**Figure 2:**
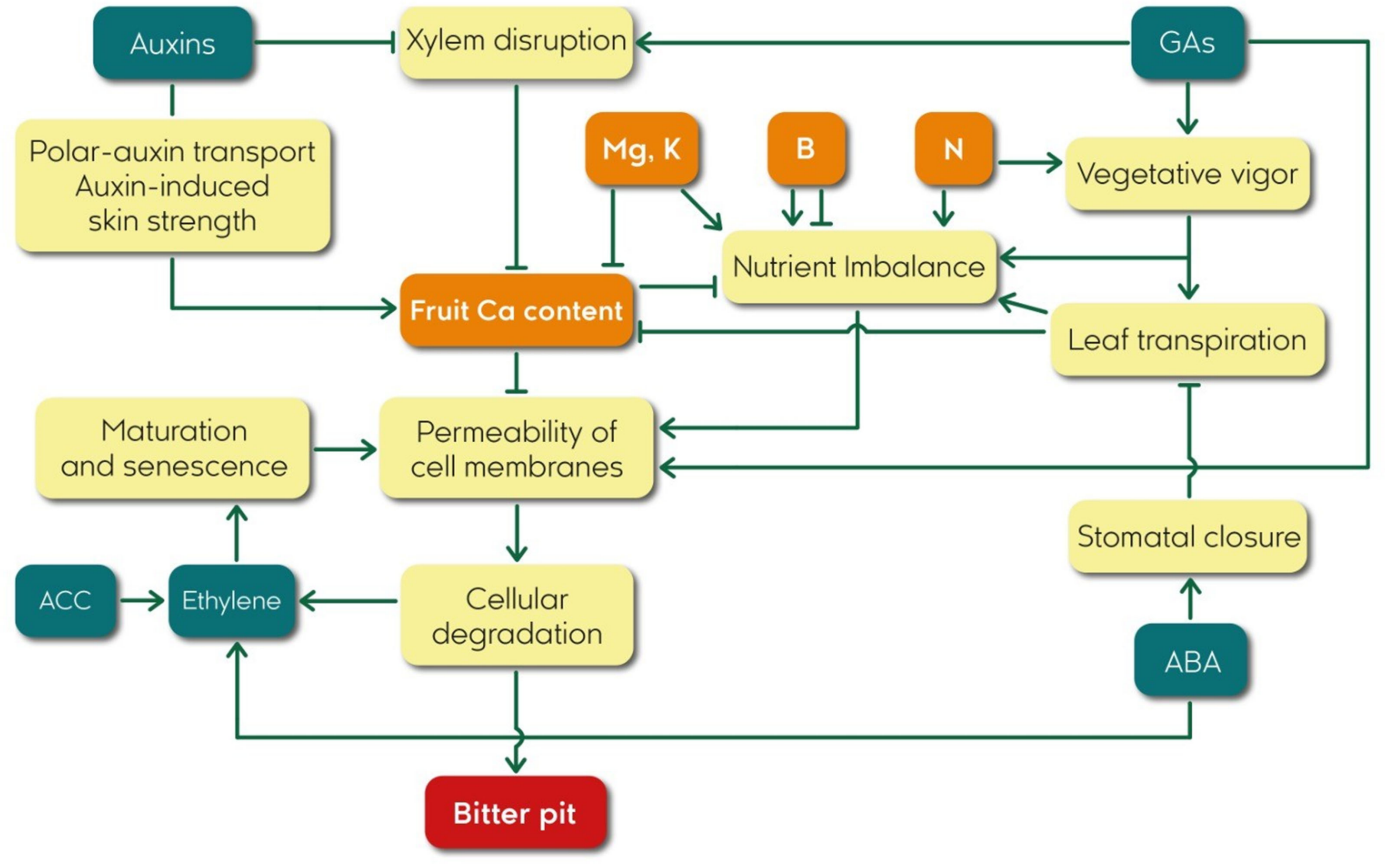
Flowchart of relationships among plant growth regulators, Ca content, and bitter pit in apple fruits.

A better knowledge of the factors that stimulate bitter pit development will help refine research hypotheses and develop more effective control strategies. The present article aims to review the relevant available research literature identifying different proposed physiological mechanisms that contribute to the development of bitter pit. We focused on the role of mineral nutrition, plant growth regulators, and other factors in the development of this disorder (**Figure 2**), providing a comprehensive overview of the state of the art in bitter pit research and highlighting the key challenges and opportunities for future research to understand the factors that influence in bitter pit occurrence and management strategies to prevent or mitigate the impact of bitter pit on apple production.

## 2 The role of calcium and its link to bitter pit incidence

### 2.1 Calcium in fruit

Bitter pit has been extensively associated with low fruit Ca content. Ca is important for fruit cell wall integrity, adhesion, and overall fruit structure (Rosenberger et al., 2004). DeLong (1936) first reported that bitter pit-affected fruit had lower Ca concentrations than healthy fruit. Since then, many publications have reported the negative relationship between Ca and bitter pit for many susceptible cultivars. However, other works have shown a weak correlation between total Ca content in fruit and bitter pit incidence, suggesting that low-level Ca content in fruit does not always result in disorder development. The association between bitter pit and low Ca content in fruit has been further supported by experiments showing increased fruit Ca content and decreased bitter pit incidence when Ca sprays are applied to the fruit surface (Van der Boon, 1980; Casero et al., 2010; Torres et al., 2017b). These clear relationships support existing hypotheses linking Ca content and bitter pit incidence, which is ultimately caused by localized cell death in fruit cortical tissue. However, Ca deficiency could be a secondary factor, increasing an existing risk of bitter pit development (Saure, 1996). Ca sprays may increase tolerance to physiological disorders by stabilizing the cell membranes, reducing their permeability, and subsequently regulating fruit respiration and ethylene production (Recasens et al., 2004).

Ca is transported to the aerial portions of the plant primarily through the xylem after it is taken up from the soil by roots in calcium ion (Ca^2+^) form, and this process is affected by transpiration. Hence, there has been a strong positive relationship reported between Ca in plant organs and transpiration (Montanaro et al., 2010; Kalcsits et al., 2019; Paiva, 2019; Carrasco-Cuello et al., 2024). Ca is mainly absorbed by the fruit during the first developmental stage, while the greatest absorption rates of other macronutrients occur later during fruit development (Casero et al., 2017). Casero et al. (2017) observed a short peak of Ca accumulation in fruit from the end of shoot growth until harvest when the competition for nutrients declines after the cessation of shoot growth. These data suggest that the periods of low leaf area (during the first fruit developmental stage) and depressed shoot growth facilitate greater Ca delivery to fruit tissues caused by reduced demand from leaf tissue relative to the supply by the roots.

Transports in the xylem occur through the apoplast, while phloem transports of photoassimilates from source leaves to sink tissues occur symplastically. Ca^2+^ has low mobility in phloem, and its levels are low in the cytosol, while the vacuole and apoplast have higher Ca concentrations (Gilroy et al., 1993; Bush, 1995). Ca in functional sieve tube elements must be in the micromolar range (10–3,000 µM), and thus, Ca^2+^ transport by phloem is not able to meet the demand of most tissues due to its low cytosolic concentrations (Paiva, 2019).

**Figure 3:**
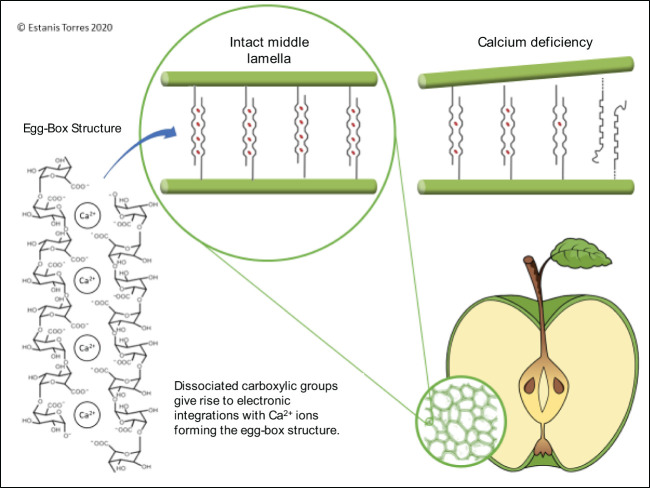
Schematic drawing of the “egg-box” model and the role that calcium plays in its stability and occurrence of calcium disorders.

Ca is involved in many processes, including cell wall formation, membrane stability, enzyme activation, and regulation of gene expression (White and Broadley, 2003). Ca supports cellular membrane integrity and function by binding to phospholipids and proteins at the membrane surface (de Freitas et al., 2010). Ca stabilizes and protects the cell walls from enzymatic degradation. The carboxyl group of pectin in the cell walls binds to Ca^2+^ to form Ca pectates, which act to generate a cementing effect between cells (Fry, 2004) (**Figure 3**). Degradation of pectates naturally occurs during ripening via polygalacturonase, which can be inhibited by high concentrations of Ca (Wehr et al., 2004). Ca deficiencies can induce the degradation of cell membranes, leading to cell death and tissue collapse, producing symptomatic bitter pit development (Ferguson and Watkins, 1989). Ferguson and Triggs (1990) reported that Ca deficiencies in cell membranes increase the permeability of acids and phenols, which penetrate easier into the cytoplasm, destroying or coagulating enzymes of mitochondria or other subcellular particles.

**Figure 4:**
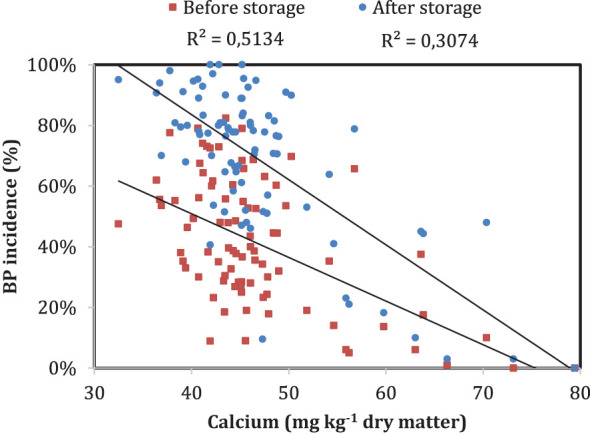
Relationship between bitter pit incidence and fruit calcium content measured 2 months before harvest in ‘Honeycrisp’ apples at the Cornell Agriculture Experiment Station in Geneva, New York (2021). Each point represents bitter pit incidence in a tree at harvest (red) and 3 months after storage (blue). The bitter pit incidence was assessed on a 100-fruit sample per tree.

Although the biochemical importance of Ca in fruit structural integrity is clear, low bulk Ca content in fruit does not always result in disorder development (Watkins, 2003). Several studies have reported weak correlation between Ca content in fruit and bitter pit incidence (Ferguson et al., 1979; Terblanche et al., 1980; Le Grange et al., 1998; Lotze et al., 2008; Torres et al., 2017b) and is also demonstrated in **Figure 4** where analyses of fruit Ca content and bitter pit incidence in 80 trees from the same orchard revealed that there was a weak relationship between both variables, especially when bitter pit was assessed after storage. Ca in fruit is present in various forms or fractions, including soluble, exchangeable, bound, and structural Ca (Jemrić et al., 2016). Therefore, fruit with similar total Ca content may have different partitioning among Ca pools (Bonomelli et al., 2018). Different Ca fractions in fruit have different roles in fruit development and quality, and their relationship with bitter pit incidence is not always clear. Soluble Ca is the fraction that is readily available within the fruit (Amarante et al., 2013), and exchangeable Ca is the fraction that is loosely bound to fruit cell walls and can be replaced by other cations (de Freitas et al., 2015). Both fractions contribute to cell membrane integrity and regulate cellular metabolism (Bonomelli et al., 2018). Unlike soluble and exchangeable Ca, bound and structural Ca fractions cannot be easily exchanged (de Freitas et al., 2015). They are tightly bound to the cell walls and are incorporated into fruit during cell growth and development (Hocking et al., 2016). These fractions are important for maintaining fruit structure, rigidity, and texture; preventing fruit deterioration; conserving moisture content; and retaining fruit freshness (Thomas and Adarsh, 2020). Some cytochemical studies have indicated that the majority of apple Ca^2+^ is found in vacuoles and cell walls of fruit for cell regulation (Harker and Venis, 1991; de Freitas et al., 2015). Some studies suggest that higher levels of these Ca fractions correspond better to lower bitter pit incidence than bulk Ca content (Perring, 1986; Harker and Venis, 1991; de Freitas et al., 2015; Jemrić et al., 2016). Some studies suggest that higher levels of these Ca fractions correspond better to lower bitter pit incidence than bulk Ca content (Perring, 1986; Harker and Venis, 1991; de Freitas et al., 2015; Jemrić et al., 2016). de Freitas et al. (2010) reported that Ca^2+^ accumulation into storage organelles and Ca^2+^ binding to the cell walls represent important contributors to bitter pit development. Later, de Freitas et al. (2015) concluded that high levels of loosely cell wall-bound or non-cell wall-bound Ca^2+^ may prevent bitter pit and that its susceptibility can be enhanced by increasing water-insoluble pectin Ca^2+^. Islam et al. (2022) observed that the tolerance of the rootstock B.10 to bitter pit could be attributed to a reduction of the amount of Ca^2+^ cross-linked with pectin and could have higher free apoplastic Ca concentrations that are essential for maintaining cell membrane integrity. In short, the cellular partitioning of Ca and its balance with other cations may affect the level of cell membrane-bound Ca as well as many metabolic processes (Thomas and Adarsh, 2020). Hence, cytoplasmic Ca regulation is important for controlling numerous metabolic pathways and/or avoiding cytotoxic responses that could lead to cell death (Paiva, 2019). However, the relationship between Ca fractions in fruit and bitter pit incidence is complex, and more research is needed to better understand the impact of each Ca fraction on bitter pit.

### 2.2 Balancing Ca with other plant nutrients

Other nutrients, such as potassium (K), magnesium (Mg), nitrogen (N), phosphorus (P), or boron (B), have been proposed to exacerbate Ca deficiency symptoms (Cheng and Sazo, 2018). In addition to absolute Ca content, its balance relative to other macro- and micronutrients has been a primary focus for plant nutrition research because of its important contributions to the development of physiological disorders (Kalcsits et al., 2020). These nutrients can affect Ca uptake, assimilation, and remobilization (Ferguson et al., 1999; Casero et al., 2004). Mg and K are well known as Ca antagonists, and high levels of these two nutrients can affect the stability and permeability of the cell membrane and lead to cell death (Buchloh and Bangerth, 1974). Contrary to Ca, Mg and K are easily transported through phloem; hence, if Ca concentrations in cell membranes are insufficient, K and Mg could take their place affecting cell membrane structure (Ferguson and Watkins, 1983; Hopfinger et al., 1984; Witney et al., 1991). Dilmaghani et al. (2005) observed a negative correlation between the relationship of K/Ca and the firmness of fruits at the time of harvest. Van der Boon (1980) and Pavičić and Miljcović (1991) observed that the incidence of bitter pit in stored apples increased with increasing (K+Mg)/Ca and K/Ca ratios. Krishkov (2007) estimated the occurrence level of bitter pit according to the concentration of K and Mg, regardless of the content of Ca. Other authors also concluded that interaction of Ca with K and/or Mg could be more involved with bitter pit than Ca individually (Sió et al., 1999; Drahorad and Aichner, 2001; Amarante et al., 2006a, b; Wińska-Krysiak and Łata, 2010; Guerra et al., 2011; Miqueloto et al., 2011; do Amarante et al., 2013, 2013; Sió et al., 2018).

The role of N in bitter pit development is more complex. Alonso et al. (2003) reported higher bitter pit incidence when N fertilization increased. Fallahi and Mahdavi (2020) recommended against using N fertilizers in susceptible orchards because they stimulate vigor and, consequently, increase the risk of bitter pit development. High N levels in the soil and/or in the tree could affect both Ca partitioning between leaves and fruit and/or dilution of Ca pools in the fruit during late-season fruit growth (Wiersum, 1974). Most studies suggest excessive vigor as the cause of N-induced bitter pit. High N fertilization rates have higher vegetative growth (Ro and Park, 2000). Vigorous shoot growth can increase whole transpiration rates, leading to the diversion of Ca supplied by root uptake to leaves instead of developing fruit (Gilliham et al., 2011). Valverdi and Kalcsits (2021) evaluated how different rootstocks affected nutrient uptake in ‘Honeycrisp’ apples and suggested that rootstock-induced vigor can contribute to nutrient imbalances in leaves and fruit that could affect the development of physiological disorders such as bitter pit. High rates of N fertilization could also increase cell expansion and greater fruit growth and, consequently, lead to a greater Ca content dissolution and susceptibility to bitter pit (Ferguson and Watkins, 1989; de Freitas et al., 2015; Kowalczyk et al., 2022). N form may be related to bitter pit occurrence, and some authors have suggested that the ammonium form of N could increase the susceptibility to bitter pit compared to nitrates since in soil ammonium suppresses the uptake of cations like Ca (Ludders, 1979; Fukumoto and Nagai, 1983; Watkins, 2003; Valverdi and Kalcsits, 2021).

B is an essential microelement with an important role in many enzymatic reactions, such as sugar transport, division and growth cell, respiration, photosynthesis, and cell wall synthesis, as well as in regulating pollen tube growth (Fang et al., 2019). B is known to play a role in the formation and stability of pectin, a major component of the fruit cell walls (Paparnakis et al., 2013). B is primarily transported through xylem vessels like Ca, and its deficiency can also contribute to various physiological disorders in fruit trees (Dixon et al., 1973; Wójcik and Cieslinski, 2000). Apple is among the most B-sensitive plants, commonly affected by either deficiency or excess of B (Paparnakis et al., 2013). According to previous literature, bitter pit is more common when B is deficient since B improves Ca movement to fruit and cell wall and supports membrane stabilization (Wójcik et al., 1999; Wójcik and Cieslinski, 2000; Rosenberger et al., 2004). Dixon et al. (1973) reported that soil applications of B improved the effectiveness of Ca sprays, increasing the percentage of non-symptomatic fruit in ‘Egremont Russet’ apples. Wójcik and Cieslinski (2000) observed that applying B after bloom increased Ca in ‘Sampion’ apples, potentially contributing to increased apple firmness after storage and decreased sensitivity to bitter pit. B also plays a crucial role in pollen germination and fruit development, and its deficiency can lead to poor root development (Fang et al., 2019), all of which are factors associated with the bitter pit occurrence. However, other results suggested the opposite, showing an increase in bitter pit when a B surplus existed (Wójcik and Cieslinski, 2000; Torres et al., 2017a). Benavides et al. (2002) observed a negative correlation between Ca and B content in ‘Golden Smoothee’ apples, which could enhance the occurrence of Ca disorders such as bitter pit. Some authors have also linked excessive B with accelerated fruit maturation (Peryea, 1994; Wójcik et al., 1999) and increased fruit disorders (Bramlage and Thompson, 1962; Martin et al., 1976; Peryea, 1994).

All these results showed that the influence of B on bitter pit remains unclear and that B requirements and responses to fertilizer applications could be dependent on the cultivar or environment (Torres et al., 2017a). Different responses to B fertilization for apples could be related to pH soil and/or climatic conditions. In areas with low soil pH values, frequent rainfalls tend to leach B relatively easily down the soil profile and deplete it from the root zone due to its non-cationic nature. Since most investigations on B have been conducted in temperate or high-rainfall areas, research efforts have mainly concentrated on problems related to B deficiency (Paparnakis et al., 2013). However, in areas with high soil pH values and where high rainfall is less frequent, like some Mediterranean regions, B would not be commonly leached from soils, and thus, plants could suffer from B excess. In these cases, this may disrupt the balance of other essential nutrients, including Ca, triggering various physiological processes in fruits, such as bitter pit.

To summarize, although B deficiency alone may not directly cause bitter pit in apples, it could significantly contribute to the disorder by compromising the structural integrity of cell walls, impairing Ca mobility, disrupting enzyme activity, and affecting hormonal regulation (Fang et al., 2019). Conversely, like many other nutrients, B must be applied within a certain range to avoid negative side effects. Factors such as soil pH, texture, and organic matter content, as well as the susceptibility of cultivars, can influence the availability of B to plants (Peryea, 1994). Therefore, it is important to consider these factors to adjust B application rates accordingly to ensure optimal plant nutrition and prevent the occurrence of physiological disorders.

It seems clear that Ca deficiencies and their content relative to the content of key micro- and macronutrients contribute greatly to fruit susceptibility to bitter pit. However, the precise mechanisms involved in its development are still not fully understood, and prognosis based only on these factors can be poor in some circumstances. This point has prompted debate about whether Ca deficiency is the key factor involved or is just a corresponding consequence of the real cause of bitter pit (Saure, 2002; de Freitas et al., 2013; Saure, 2014). In the following section, several alternative theories will be presented that may also contribute to bitter pit development but where Ca deficiency or nutrient imbalances are often also observed under these same scenarios.

## 3 The auxins as regulator of Ca transport

Auxins are key phytohormones contributing to plant growth and development through their impact on cellular elasticity and, subsequently, cell growth and elongation. There have been many studies that have delineated the controlling pathways for auxin perception, signaling, and its contribution to Ca transport (Benjamins and Scheres, 2008). Auxin regulates the mobilization of photoassimilates in source tissues (mainly leaves) and elevates the translocation of carbohydrates toward sink organs (e.g., roots and fruits, respectively) (Balasubramanian et al., 2023). The relationship between auxins and bitter pit may be related to the link between Ca absorption in plant tissue and polar auxin transport, where tissues that are low in auxins have poor absorption of Ca. Polar auxin transport is the regulated transport of auxins in plants, and among its functions, the most important is the ability to coordinate signaling controlling plant development (Balasubramanian et al., 2023). Many studies indicate that Ca-mediated processes may be involved in modulating auxin response and polar auxin transport (Li et al., 2019). Indeed, Stahly and Benson (1970) and Oberly (1973) observed in ‘Golden Delicius’ and ‘Northern Spy’ apples, respectively, lower accumulation of Ca in the fruit and higher bitter pit incidence when spraying triiodobenzoic acid (TIBA), a known inhibitor of polar auxin transport (**Table 1**). Parallel patterns in polar basipetal auxin transport and Ca transport have been observed for multiple fruit species including tomato, kiwifruit, and apple (Stahly and Benson, 1970; Banuelos et al., 1987; Sorce et al., 2011).

**Table 1:** Effect of exogenous hormonal applications on Ca content in fruit and bitter pit (BP) incidence.

Hormone	Expected physiological response	Effect	Cultivar	Reference
Abscisic acid	Transpiration reduction in leaves	↓ BP and ↑ Ca	‘Super Chief’	Gomez and Kalcsits (2020) Angmo et al. (2022)
Auxins	Improvement of fruit B content	↓ BP (IAA) ↑ BP (NAA)	‘Notaris’	Van Stuivenberg and Pouwer (1950) Mulder, 1951 cited by Griffith and Einhorn (2023)
	Vascular function stimulation	↓ BP and ↑ Ca	‘Honeycrisp’	Griffith et al. (2022)
	Flower-promoting	↓ BP and = BP	‘Honeycrisp’	Cline (2019)*
Ethylene (ethephon)	Advancement of fruit maturity	↓ BP and = Ca	‘Bramley’s Seedling’	Prinja (1990)
	Flower-promoting	↓ BP and ↑ BP	‘Honeycrisp’	Cline (2019)*
Gibberellins	Fruit and vegetative growth stimulation	↑ BP and ↓ Ca	‘Catarina’ and ‘Fuji’ ‘Braeburn’	Silveira et al. (2012) do Amarante et al. (2020)
		= BP and = Ca	‘Honeycrisp’	Serban and Kalcsits (2018)
Prohexadione-Ca	Reducing extension shoot growth	↓ BP and ↑ Ca	‘Catarina’ and ‘Fuji’ ‘Braeburn’	Silveira et al. (2012) do Amarante et al. (2020)
		↓ BP and ↑ BP = BP and = Ca	‘Honeycrisp’ ‘Honeycrisp’	Donahue et al. (2018)** Serban and Kalcsits (2018)
Paclobutrazol	Reducing extension shoot growth	↓ BP and ↑ Ca	‘Gardiner Delicious’	Greene (1991)
Triiodobenzoic Acid	Effect on Ca movement and accumulation in plant tissues	↑ BP and ↓ Ca	‘Golden delicious’ ‘Northern Spy’	Stahly and Benson (1970) Oberly (1973)

IAA, indole-3-acetic acid; NAA, 1-naphthaleneacetic acid.

^*^ Three sprays of NAA or ethephon reduced BP incidence, while six sprays increased it.

^**^ Prohexadione-Ca applications at pink button stage reduced BP incidence, while petal fall increased it.

Indole-3-acetic acid (IAA) is the most common naturally occurring auxin. IAA is produced in seeds, in the apical meristems of shoots, stems, and roots from transamination and decarboxylation reactions of tryptophan (Griffith and Einhorn, 2023; Luckwill, 1953; Van der Krieken et al., 1993). Hertel (1983) and Banuelos et al. (1987) related the appearance of bitter pit to the low auxin concentrations in fruit. Hertel (1983) suggested that IAA transport can preferentially stimulate the release of Ca from vacuoles or cell walls to the cytoplasm, increasing cytoplasmic Ca concentrations. This increase of cytoplasmic Ca would be associated with stimulation of synthesis or renewal of cell wall material and, consequently, with a reduction of the degradation and risk of bitter pit. Banuelos et al. (1987) reported that endogenous IAA controls Ca transport mechanisms, independent from transpiration flow, by increasing sink strength in those specific tissues. Additionally, intercellular Ca mobilization across very short distances occurs through the auxin pump mechanism. According to Banuelos et al. (1987), lower auxin content could limit Ca transport through these two pathways, contributing to bitter pit development in apple fruit.

Exogenous auxin applications were proposed to reduce bitter pit but with irregular results (**Table 1**). Van Stuivenberg and Pouwer (1950) hypothesized that auxin concentrations must exceed a specific threshold to overcome B deficiencies and prevent the development of cork spot and bitter pit (Griffith and Einhorn, 2023). Indeed, they suggested that disorder incidence could be controlled by spraying the trees with IAA at the end of June or in the first half of July (northern hemisphere) during the cell expansion phase of fruit growth. However, another study where the IAA was supplemented by the synthetic auxin 1-naphthaleneacetic acid (NAA) reported increased bitter pit incidence in NAA-treated trees (Mulder, 1951 cited by Griffith and Einhorn, 2023). Cline (2019) also obtained different responses to pre-harvest NAA applications depending on the number of applications (**Table 1**). According to these results, the relationship between auxins and bitter pit incidence may vary depending on several factors, such as differences in growing conditions, weather, and soil (Cline, 2019; Griffith and Einhorn, 2023).

Overall, it appears evident that auxins may affect bitter pit development in apples. However, the exact mechanisms underlying the relationship between auxins and bitter pit in apples are not fully understood. In addition to the connection between Ca absorption and polar auxin transport, auxins could also contribute to xylem disruption (Jacobs, 1952; Johnsson et al., 2019). It is widely known that Ca is translocated from the root system to the shoot and fruits via the xylem; hence, some authors have worked on the role of auxins in the differentiation and timing of xylem development and its relationship with the bitter pit (Griffith et al., 2022).

## 4 Xylem vessel disruption

Xylem functionality has been associated with bitter pit (Griffith and Einhorn, 2023). Since Ca movement from roots to above-ground tissues follows the transpiration stream, xylem connectivity and continued flow into developing fruit are important for Ca accumulation to occur (Hocking et al., 2016). Xylem vessel functionality naturally decreases during fruit growth and development (Dražeta et al., 2004; Miqueloto et al., 2014). This xylem function decays relatively early in the season and mirrors the decline in Ca import (Wilkinson, 1968; Jones et al., 1983; Lang, 1990; Casero et al., 2017).

Dražeta et al. (2004) suggested that decreases in xylem functionality are naturally programmed to reduce diurnal apoplastic backflow of solutes from fruit to the tree during late fruit development. The direction of xylem movement can reverse course during diurnal cycles in apples such that flow is negative into the fruit during the day and positive at night (Lang, 1990; Lang and Volz, 1998). Hence, xylem dysfunction may be a response to minimize the outflow of xylem sap from the fruit but may also come at the expense of reducing the import of xylem‐borne minerals, such as Ca, to the fruit. According to Dražeta et al. (2004), xylem dysfunction begins earlier in the season for apple cultivars that are more susceptible to bitter pit. Other studies have also investigated the relationship between xylem vessel disruption and bitter pit in apples (Amarante et al., 2012; Miqueloto et al., 2014; Angmo et al., 2022). According to Song et al. (2018), xylem conductivity is also determined by xylem anatomic features in the pedicel, which is the site where xylem functionality is lost. All these studies found that fruit with bitter pit had a higher incidence of disrupted xylem vessels compared to fruit without bitter pit. This has led to the hypothesis that xylem disruption could affect Ca uptake and, consequently, trigger bitter pit occurrence as a result of a Ca deficiency in the fruit.

There are two competing theories on the cause of losses to xylem functionality in fruit: 1) mechanical damage to xylem vessels creating permanent losses to functionality and 2) a shift from xylem water transport to phloem water transport from carbohydrate loading into fruit leading to reversible changes in xylem functionality (Dražeta et al., 2004). Losses in xylem functionality may be associated with an increase in the number and/or elongation of parenchyma cells, which compresses xylem vessels without affecting the functionality of phloem vessels. This would cause a decline in Ca import flow to the fruit but would not affect K, Mg, and N flow, resulting in higher susceptibility to bitter pit (Lang and Ryan, 1994; Amarante et al., 2013; Miqueloto et al., 2014). Miqueloto et al. (2018) investigated the mechanisms that regulate fruit Ca content and susceptibility to bitter pit in two cultivars with low or high susceptibility to bitter pit (‘Fuji’ and ‘Catarina’, respectively). They found that losses to xylem functionality, reductions in lenticel pore area, and increases in pectin methylesterase (PME) activity in the peel tissue may all contribute to reductions in fruit Ca content at the distal portions of the fruit and increase bitter pit incidence. The disruptions in the xylem vessels may also further affect the transport of water and other nutrients from the root system. When xylem functionality decreases, water and nutrient transport must occur through the phloem. These changes to transport dynamics may affect the fruit gas exchange and lenticel functionality. Water transport and lenticel functionality are critical for gas exchange and regulating fruit temperature (Venturas et al., 2017). Hence, xylem vessel degradation could increase the risk of hypoxia and temperature stress, stimulating cell death in affected tissues and, consequently, the bitter pit symptoms. Hypoxia and temperature stress promote the increase of reactive oxygen species (ROS), leading to oxidative stress (Pucciariello and Perata, 2017), which is implicated in different physiological disorders suffered by various horticultural crops related to Ca deficiency (Saure, 2014).

The composition and relative abundance of auxins within fruit tissue could have a significant role in xylem disruption during fruit growth (Miqueloto et al., 2014). Saure (2005), and more recently Griffith and Einhorn (2023), proposed that the correlative relationships between bitter pit and nutrient concentrations within fruit tissues may be merely consequences of hormonal imbalances, especially notably auxins, gibberellins, and abscisic acid, that facilitate xylem dysfunction and, consequently, lead to imbalances in nutrient supplies to fruit. Griffith et al. (2022) hypothesized that strategies that limit mechanical xylem vessel damage may help to improve Ca transport to fruit and, consequently, prevent the development of bitter pit (**Table 1**). They tested this working hypothesis and observed that three successive applications of 20 ppm IAA at 30 days, 45 days, and 60 days after full bloom slowed the development of xylem dysfunction and decreased bitter pit incidence in ‘Honeycrisp’ apples by 65%.

## 5 The effect of gibberellins on Ca mobility and bitter pit

Saure (1996) hypothesized that an increase of gibberellins in fruit may be an underlying cause for bitter pit. This theory was based on the observation that sound fruit without bitter pit symptoms can sometimes have extremely low Ca fruit concentrations. Following this, cell permeability could increase with increasing gibberellins and with it the risk of dehydration and cellular degradation. Conforming to this hypothesis, Ca could play a secondary role where apples with a high Ca content have more stable membranes that resist degradation. Elevated concentrations of gibberellins in fruit could also have consequences on xylem formation. Gradual reductions in auxin concentration and a concurrent increase in the concentration of active gibberellins may constitute a signal for cells to transition from an expansive phase to a maturation one or disruption of xylem (Immanen et al., 2016; Johnsson et al., 2019). Therefore, high levels of gibberellins in fruit may accelerate xylem dysfunction and, consequently, contribute to the development of bitter pit through mechanisms described earlier.

Furthermore, gibberellins play a key role in the stimulation of fruit and shoot growth. Vigorous growth often coincides with high levels of physiologically active gibberellins in the tissues (Saure, 2005). Rapid fruit and shoot growth correlate positively with bitter pit susceptibility through changes in Ca distribution to fruit and diluting Ca as fruit growth increases. Therefore, high gibberellin content during periods of vigorous growth could have both direct and indirect effects that stimulate the development of bitter pit (Saure, 2005). Supporting this hypothesis, Greene (1991), Silveira et al. (2012), and do Amarante et al. (2020) reported a reduction in bitter pit when trees were treated with gibberellin synthesis inhibitors (**Table 1**). Greene (1991) reported that ‘Gardiner Delicious’ apple trees treated with paclobutrazol had lower vegetative vigor, higher Ca fruit content, and lower bitter pit incidence. Silveira et al. (2012) observed either a reduction or an increase in bitter pit in ‘Catarina’ and ‘Fuji’ apple trees when prohexadione-Ca and gibberellin GA_3_ were applied, respectively. do Amarante et al. (2020) also reported that ‘Braeburn’ apple trees treated with GA_4 + 7_ showed a higher incidence of bitter pit in fruit than trees treated with prohexadione-Ca. These three studies demonstrate the negative effects that gibberellins can have on bitter pit. However, Serban and Kalcsits (2018) and Donahue et al. (2018) did not observe changes in fruit Ca concentrations or bitter pit incidences by applying GA_3_ or prohexadione-Ca after full bloom in young ‘Honeycrisp’ apple trees. These results highlight a need for further studies to identify the factors explaining the relationship between gibberellins and bitter pit.

## 6 Interactions between abiotic stress and bitter pit

Abiotic stress refers to the negative impact of non-living factors on plants and includes drought-, heat-, or soil-based nutrient deficiencies. An increase in intracellular Ca^2+^ concentrations is a common event in most stress-induced signal transduction pathways (Xu et al., 2022). Ca^2+^ is an important second messenger for understanding plant–abiotic stimulus interactions (Van Zelm et al., 2020). However, when apple trees experience soil-based abiotic stress, root activity and Ca uptake from the soil may decline, leading to deficiencies in the fruit (Fallahi, 2012). For example, nutrient accumulation in the biomass of woody plants can decrease under drought conditions as transpiration declines, particularly for those that depend on transpiration such as Ca, Mg, Fe, and S (Sardans et al., 2008). This can be related to several factors including a decrease in soil moisture that reduces soil mass flow and diffusion of nutrients, a decrease in root growth, and a decrease in photosynthetic capacity, sap flow, and transpiration (Sardans et al., 2008). These factors have a cascading effect on plant growth affecting overall nutrient demand and changing nutrient balance within the tree.

Abscisic acid (ABA) is a critical hormone regulating plant response to abiotic stress. ABA is synthesized in response to various environmental stresses (Finkelstein et al., 2002), including drought (Zhang et al., 2019), high salinity (Wang et al., 2022), extreme temperatures (Yang et al., 2011), and other factors that can disrupt normal plant growth and development. One of the primary functions of ABA is to regulate stomatal closure, which helps to reduce water loss from the plant during times of drought or high temperatures. Overall, ABA helps plants tolerate abiotic stresses by regulating water use, enhancing stress tolerance, and activating stress-responsive genes and pathways. The role of ABA on bitter pit incidence identified in the literature has attributed its effect on leaf stomatal closure and reducing transpiration to the distribution of Ca to fruit and leaves (Falchi et al., 2017). ABA was reported to reduce blossom end-rot in tomato and is a disorder analogous to bitter pit in apple (de Freitas et al., 2011). For a study using ‘Super Chief’ apple cultivar, Falchi et al. (2017) revealed that four foliar sprays with ABA during the season (starting from 66 days after full bloom) can be effective in apple bitter pit prevention via a preferential supply of Ca^2+^ to fruit. They hypothesized that bitter pit reduction could be associated with the increased Ca^2+^ concentration in the apoplast and a coordinated regulation of specific Ca^2+^-related genes.

Saure (2014) implicated abiotic stress as a cause of physiological disorders suffered by various horticultural crops related to Ca deficiency. According to this author, Ca deficiencies observed when disorders such as bitter pit are present could be the result of stress and not the cause of the disorder. Supporting this hypothesis, stress increases the production of ROS associated with lipid peroxidation and an increase in the leakiness of membranes, leading to rapid vacuolation of parenchyma cells and the loss of ions, including water-soluble apoplastic Ca^2+^. Prior to Wińska-Krysiak and Łata (2010); Saure (2014) investigated the relationship between the development of bitter pit and the activity of the enzyme lipoxygenase (LOX), an enzyme involved in fruit respiration. They concluded that apple cultivars with higher LOX activity are more stressed and more susceptible to bitter pit. Supporting this, Krawitzky et al. (2016) identified several types of proteins related to the responses to oxidative stress induced in fruit affected by bitter pit. Pathogenesis-related proteins are expressed in biological systems as a defense response to abiotic stress (Pühringer et al., 2000). These proteins may be synthesized in response to several stress factors during bitter pit development and may be expressed even before external symptoms are visible in the skin tissues (Krawitzky et al., 2016).

Abiotic stress does not always stimulate bitter pit development. Reid and Kalcsits (2020) reported that bitter pit incidence was lower for trees that were exposed to water limitations in a semi-arid environment. Strong reductions in stem water potential were observed, and bitter pit was the lowest in trees where abiotic stress was imposed during the summer when water demand was the greatest. In this case, water limitations were used to limit fruit expansion and increase fruit density, which reduced Ca dilution often observed in these cases (Kalcsits et al., 2020). Therefore, the timing of the abiotic stress is important. If it occurs at a point in development where Ca development is high and uptake is reduced, then the results would be expected to be negative. If stressful conditions reduce fruit growth or vigor, then it may improve the distribution and reduce dilution of Ca that regularly occurs during the season in susceptible cultivars (Casero et al., 2017). Nevertheless, there is a need for further research to separate the conflicting factors associated with abiotic stress on root activity, overall tree physiology, and fruit biochemistry to better understand environmental factors that contribute to bitter pit development.

## 7 Other causes and factors of bitter pit

Bitter pit development is a complex process influenced by multiple factors in addition to those indicated here, including cultivar (Ferguson and Watkins, 1989), rootstock (Lordan et al., 2019), irrigation (Reid and Kalcsits, 2020), fruit maturity (Prange et al., 2011), and storage conditions (Watkins et al., 2004). Horticultural management affecting crop load and vegetative vigor can also have strong effects on bitter pit development (Serra et al., 2016). However, the direct connections between these management strategies and planting decisions still need to be more closely studied to identify optimum combinations that lead to the consistent production of fruit with lower bitter pit incidence (Baugher et al., 2017). The effect of other classes of hormones and plant growth regulators such as cytokinins, ethylene, jasmonates, and salicylic acid also suggest the potential for bitter pit mitigation; however, their link to bitter pit is not yet clear (Griffith and Einhorn, 2023).

Another hypothesis, suggested by Steenkamp et al. (1983), is the mechanisms of metabolization of toxic substances generated during photosynthesis, such as oxalic acid and citric acid (Steenkamp et al., 1983). Plants do not have mechanisms to extrude these toxic compounds and instead can metabolize them through the formation of insoluble salts with Ca^2+^. When the Ca content in the cell would not be enough to metabolize these acids, cell death would occur, resulting in bitter pit symptoms. From this perspective, we suggest that the disruptions in xylem vessels may also affect the photosynthesis and mechanisms that eliminate the toxic substances and/or Ca availability to metabolize the acids; however, this hypothesis has yet to be thoroughly tested. Steenkamp et al. (1983) observed high oxalate and citrate concentrations in bitter pit areas, and by vacuum infiltration, they identified deterioration of the middle lamella of fruit cells, similar to those observed in naturally occurring pits. From these results, Val et al. (2006) used ammonium oxalate cortical injections to trigger like-bitter pit symptoms and study the polypeptide pattern in symptomatic and asymptomatic fruits. Finally, they found the novel 18-kDa protein both in natural bitter pit spots and in chemically induced corky lesions. They suggested that the novel protein may be an inhibitor of pectin methylesterase, a small heat-stress protein. More recently, Zupan et al. (2013) found that higher levels of many phenolics, including chlorogenic acid and catechin, were observed in peel and cortex with bitter pit symptoms, and higher levels of hydroxycinnamates and flavonols were found in the peel immediately above bitter pit symptoms; in contrast, flavonols and anthocyanins were higher in healthy peel (Zupan et al., 2013). These metabolic profile changes could be linked to sun stress (McTavish et al., 2020). Indeed, the bagging of apples provides protection from high-intensity sunlight, and bagging apples alters metabolic profile, which helps prevent bitter pit-like symptoms (Sharma et al., 2014; Sheick et al., 2023).

Not surprisingly, bitter pit is under some degree of genetic control in fruit. Apple breeders and geneticists have tried to understand the relationship between genetic variability and bitter pit occurrence to generate bitter pit-resistant cultivars (Volz et al., 2006; Buti et al., 2018; Mao et al., 2021). Volz et al. (2006) identified a genetic component to bitter pit incidence, and this effect was present irrespective of the use of several cropping factors, average fruit weight, or even fruit mineral concentrations. For any one seedling, the family had the largest effect on bitter pit incidence followed by site and Ca content in fruit, while the harvest date had the smallest effect. Although Ca concentration also showed a strong genetic component, no relationship was found between genetic effects related to bitter pit incidence and Ca concentration. However, fruit Ca concentration is usually an important predictor of within-family variation in pit incidence. These results suggest that fruit Ca concentration may be useful as an indirect indicator index for bitter pit within, but not among families (Volz et al., 2006). More recently, Buti et al. (2018) obtained two candidate genes (MDP0000263725 and MDP0000300083) for the control of bitter pit symptom expression between susceptible and non-susceptible genotypes in the population. The authors hypothesized the ability of the fruit to regulate cell turgor and increase cell wall elasticity during fruit development in the reduction of bitter pit symptoms. Contrariwise, an inability to respond in this way to cell expansion during fruit development could lead to a higher incidence of bitter pit symptom expression in bitter pit-susceptible lines.

Apple rootstocks have different influences and effects on the nutrition and vigor of the canopy, which have been implicated in the physiology of bitter pit (Lordan et al., 2019). Donahue et al. (2021) concluded that apple rootstocks have an influence on the nutritional status of the tree canopy, which is implicated in the physiology of bitter pit and, therefore, its occurrence. Islam et al. (2022) demonstrated that rootstock selection significantly affects bitter pit incidence and can also modulate cell wall structure and Ca^2+^ homeostasis by affecting pectin de-esterification rate and pectin methylesterase activity in fruit skin and flesh tissues. This situation in the cell walls increases Ca^2+^ in the binding sites and reduces Ca^2+^ in the apoplast and could cause cell membrane breakdown. Hence, they suggested that bitter pit sensitivity could be attributed to high pectin methylesterase enzyme activity and increased pectin de-esterification rate.

Trees with different levels of crop load can show different levels of bitter pit incidence (Serra et al., 2016) possibly caused by differences in fruit growth, maturation rate, or hormone synthesis. Fruit size and maturation are important factors affecting bitter pit incidence, which, at the same time, are also influenced by crop load and irrigation. According to de Freitas and Mitcham (2012), cultivars with higher fruit senescence and ethylene production at harvest had fruit with lower Ca content and, consequently, more bitter pit. Irrigation management can also affect tree vigor and fruit growth rates. For example, Reid and Kalcsits (2020) concluded that reducing irrigation at critical phases of fruit expansion can reduce bitter pit incidence.

## 8 Conclusions

Despite bitter pit being exhaustively investigated for more than a century, the mechanisms involved in its development are still not clearly known. This is most likely due to the interrelatedness of responses that have been previously observed. However, in the past decade, significant progress has been made in understanding the biochemical changes associated with bitter pit, key factors regulating its development, and management strategies that reduce it. Even so, there is no single management option to control bitter pit, just as there is no single cause for its development. Indeed, a precise management option for bitter pit control should consider multiple factors such as cultivar, rootstock, training system, climate/meteorology, and soil, as well as the economic implications. Ca clearly corresponds to bitter pit occurrence, but the cause of localized deficiencies is minimally corrected through improved Ca fertilization. The different theories that suggest causes of bitter pit in the literature are not necessarily contradictory even though they can be inconsistent most likely due to complex interactions at whole plant and cellular scales. These theories are often complementary, and all of them share, in a direct way, Ca nutrition as a common response. However, it is not clear whether it is a consequence or an underlying cause. Emerging evidence from this review suggests that Ca deficiencies should be seen as a symptom related to complex processes and not viewed simply as a diagnosis of bitter pit risk. Nevertheless, given the strong relationship between Ca content and balance with other nutrients in fruit and bitter pit within cultivars, it still retains value as one of the most reliable predictors of bitter pit occurrence.

## Author contributions

ET: Conceptualization, Investigation, Writing – original draft, Writing – review & editing. LK: Investigation, Writing – review & editing. LG: Investigation, Writing – review & editing.
